# Cartilage organoids for cartilage development and cartilage-associated disease modeling

**DOI:** 10.3389/fcell.2023.1125405

**Published:** 2023-01-30

**Authors:** Weiping Lin, Min Wang, Liangliang Xu, Micky Tortorella, Gang Li

**Affiliations:** ^1^ Centre for Regenerative Medicine and Health, Hong Kong Institute of Science & Innovation, Chinese Academy of Sciences, Hong Kong, Hong Kong SAR, China; ^2^ The Fifth Affiliated Hospital of Guangzhou Medical University, Guangzhou, China; ^3^ Westlake Laboratory of Life Sciences and Biomedicine, Hangzhou, China; ^4^ The First Affiliated Hospital of Guangzhou University of Chinese Medicine, Guangzhou University of Chinese Medicine, Guangzhou, China; ^5^ Drug Discovery Pipeline at the Guangzhou Institutes for Biomedicine and Health, Chinese Academy of Sciences, Guangzhou, China; ^6^ Musculoskeletal Research Laboratory, Department of Orthopaedics & Traumatology, Faculty of Medicine, The Chinese University of Hong Kong, Prince of Wales Hospital, Hong Kong SAR, China; ^7^ Stem Cells and Regenerative Medicine Laboratory, Li Ka Shing Institute of Health Sciences, The Chinese University of Hong Kong, Prince of Wales Hospital, Hong Kong SAR, China; ^8^ Shenzhen Research Institute, The Chinese University of Hong Kong, Shenzhen, China

**Keywords:** cartilage organoids, LGR5, mini-joint, regenerative therapy, stem cells

## Abstract

Cartilage organoids have emerged as powerful modelling technology for recapitulation of joint embryonic events, and cartilage regeneration, as well as pathophysiology of cartilage-associated diseases. Recent breakthroughs have uncovered “mini-joint” models comprising of multicellular components and extracellular matrices of joint cartilage for development of novel disease-modifying strategies for personalized therapeutics of cartilage-associated diseases. Here, we hypothesized that LGR5-expressing embryonic joint chondroprogenitor cells are ideal stem cells for the generation of cartilage organoids as “mini-joints” *ex vivo* “in a dish” for embryonic joint development, cartilage repair, and cartilage-associated disease modelling as essential research models of drug screening for further personalized regenerative therapy. The pilot research data suggested that LGR5-GFP-expressing embryonic joint progenitor cells are promising for generation of cartilage organoids through gel embedding method, which may exert various preclinical and clinical applications for realization of personalized regenerative therapy in the future.

## 1 Introduction

Cartilage remains among the most difficult tissues to regenerate, and integration of an implant with the surrounding tissue is also a major challenge in cartilage regeneration (Huey et al., 2012; Trengove et al., 2022). Regeneration of calcified cartilage regions is also a critical issue for stable and functional integration to subchondral bone besides cartilage–cartilage integration in the field of regenerative medicine and tissue engineering.

Organoids are self-assembling three-dimensional tissues containing multiple types of cell clusters that generated from pluripotent stem cells or adult stem cells, providing a powerful tool for developmental biology and disease modeling of various tissue and organ systems *in vitro* (Dutta et al, 2017; Hu et al, 2018). Originally, organoid technology mainly comprises of gel encapsulation method that developed by Hans Clevers lab, and air-liquid interface method developed by Calvin J. Kuo lab. To date, organoids have been successfully established from adult stem cells of multiple healthy and diseased tissues and organs, including stomach (Engevik et al., 2019; Murakami et al., 2021), colon (d'Aldebert et al., 2020), intestine (Gjorevski et al., 2016; Hirota et al., 2021), lung (Miller et al., 2019; Lim et al., 2023), liver (Vyas et al., 2018), kidney (Takasato et al., 2016; Yuan et al. 2022), pancreas (Broutier et al., 2016), ovary (Kopper et al., 2019), brain (Luo and Li, 2021; Luo et al., 2022), and prostate (Huang et al., 2021) *ex vivo*.

## 2 Advancements of cartilage organoids

The development of cartilage organoid technology as useful modelling tools and robust research platforms enables the definition and disease modelling of cartilage-tissue structures *ex vivo* to facilitate drug screening through identification of key signaling pathways, and recapitulation of developmental events during joint embryogenesis and cartilage regeneration, dynamics of stem cell chondrogenic differentiation, and aging-induced degenerative joint diseases “in a dish” (Clevers, 2016; Lacko and Chen, 2019; O'Connor et al., 2021; Rothbauer et al., 2021; Sun et al., 2021). In the early 1990s, C. Schröter-Kermani and his colleagues successfully established an *ex vivo* model of a prolonged, but almost identical of chondrogenesis events *in vivo* prior to endochondral mineralization, providing a useful tool for investigations on cartilage differentiation, maturation, and degeneration (Schroter-Kermani et al., 1991). Further research by Irie, Yutaka, et al. developed sheet-shaped organoids (organoid-sheet) of cartilage-like tissues, in which cells formed multicellular aggregates (organoids), through an effective cartilage-formation method (Irie et al., 2008). Cell clusters called spheroids exert promising therapeutic potential for cartilage tissue engineering research as building blocks (Baptista et al., 2018; Kronemberger et al., 2020). Intriguingly, recent breakthroughs have uncovered “mini-joint” models comprising of multicellular components and extracellular matrices of joint cartilage for potential realization of novel disease-modifying strategies for personalized therapeutics of cartilage-associated diseases (Delplace et al., 2021; Abraham et al., 2022). A recent striking study has developed a novel differentiation protocol that generated self-organizing craniofacial cartilage organoids from human embryonic stem cells *via* a neural crest cell intermediate (Foltz et al., 2021).

Cartilage organoids are specific three-dimensional and functional cartilage-like tissues through self-assembled reconstruction of chondrocytes or chondroprogenitor cells (Irie et al., 2008; Schon et al., 2017; Gryadunova et al., 2021), which is of essential clinical significance for tremendous translational applications to repair various cartilaginous structures throughout the body, as well as organoid biobanking, disease modeling, drug toxicity testing, personalized regenerative therapy, host–microbe interaction studies, and omics analysis (including transcriptomics, proteomics, epigenomics, and metabolomics) (Dutta et al., 2017). Cartilage organoids have been successfully generated both from induced pluripotent stem cells or mesenchymal stem cells (Li Z. et al, 2022). Cartilage organoid formation and their assembly into neo-hyaline-cartilage have paved a new way for large scale cartilage regeneration such as for entire joint surfaces (Crispim and Ito, 2021). And the development of cartilaginous organoids has been applied to diverse implications in preclinical research during recent years (Table 1).

**TABLE 1:** Advancements of cartilage organoid research.

References	Cell source	Experimental model	Therapeutic outcome and mechanisms
Zimmermann et al (1990)	Embryonic mouse limb bud mesenchymal cells	Organoid culture and co-cultures *ex vivo*	Osteoblastic cells induce endochondral mineralization, whereas fibroblast-like cells inhibit this mineralization *via* soluble factors
Leijten et al (2016)	Human periosteum derived stem cells (hPDCs)	Subcutaneous implantation in nude mice	Integration of microenvironment of cellular condensation into biomaterials by encapsulating microaggregates of a hundred hPDCs induced decreased stemness-related markers and upregulation of chondrogenic genes and improved cartilage tissue formation *in vivo*
Nilsson Hall et al (2020)	Human-periosteum-derived cells	Critical-sized long bone defect in immunodeficient mice	The assembly of multiple callus organoids into an easy-to-handle scaffold-free implant resulted in full bridging of bone defects by the formation of cortical-like bone tissue with a medullary cavity containing bone marrow with the absence of fibrous tissue
Crispim and Ito, (2021)	Nucleus pulposus tissue-derived chondrocytes	A 3D suspension culture system of organoid *ex vivo*	*In vitro* neocartilage production *via* chondrocyte expansion, organoid formation, and their assembly into neohyaline-cartilage
Tam et al (2021)	Human pluripotent stem cells	Critical size long bone defects in immunocompromised mice	IL-1β accelerates bone healing by potentially increasing cartilage matrix degradation through MMP13
Hall et al (2021)	Human iPSC-derived chondrocytes/cartilage microtissues	Subcutaneous implantation in nude mice	Assembled iPSC-derived cartilage microtissues in combination with the pre-hypertrophic cartilage organoids (*IHH*, *COLX*) could form dual tissues consisting of i) a cartilaginous safranin O positive and ii) a bony osteocalcin positive region upon subcutaneous implantation
Li et al (2021)	Human induced pluripotent stem cells (hiPSC)	G-Rex 100 bioreactor culturing *in vitro*	Long-term culture of hiPSC-derived multi-tissue organoids (MTOs) results in the spontaneous emergence of mesoderm-derived articular cartilaginous tissues and MTOs cartilage resembles fetal limb bud and growth plate chondrocytes

**FIGURE 1:**
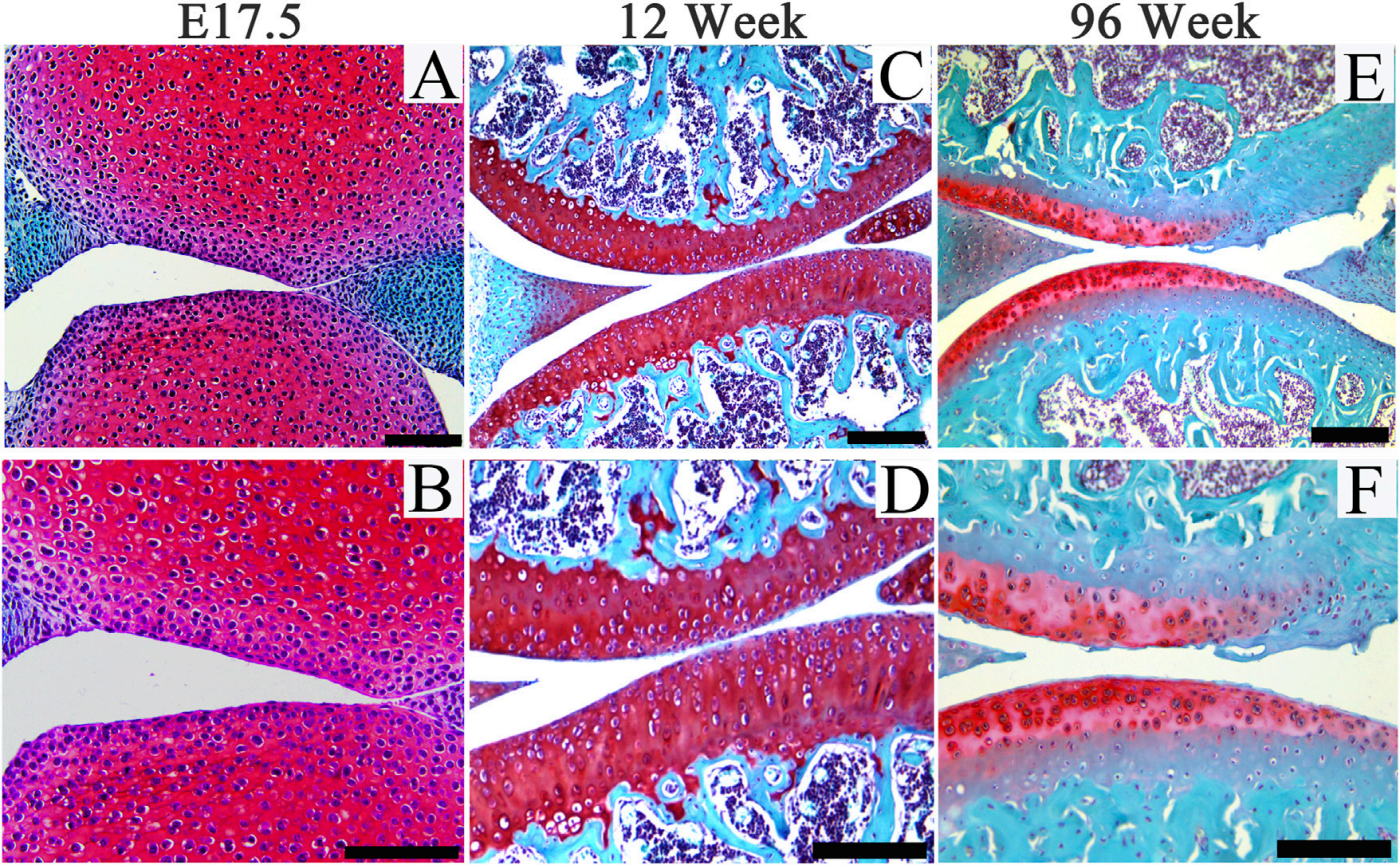
Representative images of Safranin O staining of articular cartilage of joints from embryos (E17.5), adult (12-week-old) and aging (96-week-old) mice. Scale bars = 200 μm.

Hyaline cartilages, fibrocartilages and elastic cartilages play multiple roles throughout human body including bearing loads in articular joints and intervertebral discs, providing joint lubrication, forming the external ears and nose, supporting the trachea, and forming the long bones during development and growth. Challenges associated with cartilage diseases include poor understanding of the etiology and pathogenesis and diagnostics due to the aneural and avascular nature of adult cartilages, and very limited chondroprogenitor cells within adult joint cartilage.(Krishnan and Grodzinsky, 2018; Bielajew et al., 2020; Liao et al., 2021; Lin et al., 2022). Age is a main risk factor for the development of rheumatoid arthritis, which is associated with accelerated immune aging and dysfunction of aging stem cells (Weyand and Goronzy, 2004; Goronzy et al., 2013; Weyand et al., 2014). Generally, joint cartilage usually degenerates spontaneously in elderly mammalians (Figure 1). As mitochondrial dysfunctions and age-associated systemic chronic inflammation (also termed as “inflamm-aging”) have been demonstrated linked to the development of diverse aging-associated degenerative diseases (Franceschi and Campisi, 2014; Laforge et al., 2016; Sanada et al., 2018; Josephson et al., 2019; Shin et al., 2022). To further decipher dynamic alterations of cellular and mitochondrial behaviors and structures (Tran-Khanh et al., 2005; Labbe et al., 2014; Guilak et al., 2018; Akatsu et al., 2019), and key signaling pathways involved in the interplay between mitochondrial remodeling and “inflamm-aging” may further advance the understanding of the pathophysiology of aging-associated cartilage degeneration.

## 3 Involvements of GPCRS in joint embryogenesis and cartilage pathophysiology

Leucine-rich repeat-containing G protein-coupled receptors 4–6 (LGR4–LGR6) are receptors for R-spondins, potent Wnt agonists that exert profound trophic effects on Wnt-driven stem cells compartments. The crystal structure of LGR5 has been discovered (Peng et al., 2013).

Notably, increasing evidence has demonstrated critical involvements of GPCRs during development and tissue homeostasis and regeneration in various tissue and organ systems (Luo et al., 2009; Cui et al., 2014; Feng et al., 2019; Montgomery et al., 2019; d'Aldebert et al., 2020; Lee et al., 2021; Li et al., 2022; Khedgikar et al., 2022). Crucial involvements of GPCRs, such as LGR5, in both embryonic joint development (Feng et al., 2019), and postnatal joint development in juvenile mammals (Zhou et al., 2018), as well as progression of arthritis development (Li R. et al, 2022), have been identified, suggesting targeted modulation of GPCRs on cartilage as potential novel therapeutics for arthritis management.

**FIGURE 2:**
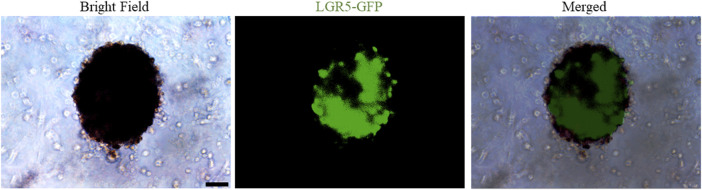
Representative images of bright-field and fluorescence of LGR5^+^-embryonic joint progenitors-based cartilage organoids formation. Scale bars = 100 µm.

Interestingly, a recent breakthrough by Rothbauer, M. et al. has successfully established microfluidic joint-on-a-chip organoid system to investigate reciprocal cross-talk between individual synovial and chondral organoids on tissue-level for modelling of arthritic diseases (Rothbauer et al., 2021). And our ongoing research suggest that LGR5-GFP^+^ embryonic joint progenitors embedded within hydrogels enable the generation of organoid-structures under appropriate culture conditions with expression of LGR5-GFP signal (Figure 2), suggesting that LGR5-expressing joint chondroprogenitor cells are potential ideal cells for cartilage-like organoids formation, disease modelling for cartilage-associated diseases, drug screening and cartilage regeneration for realization of personalized medicine.

## 4 Conclusions and future perspectives

**FIGURE 3:**
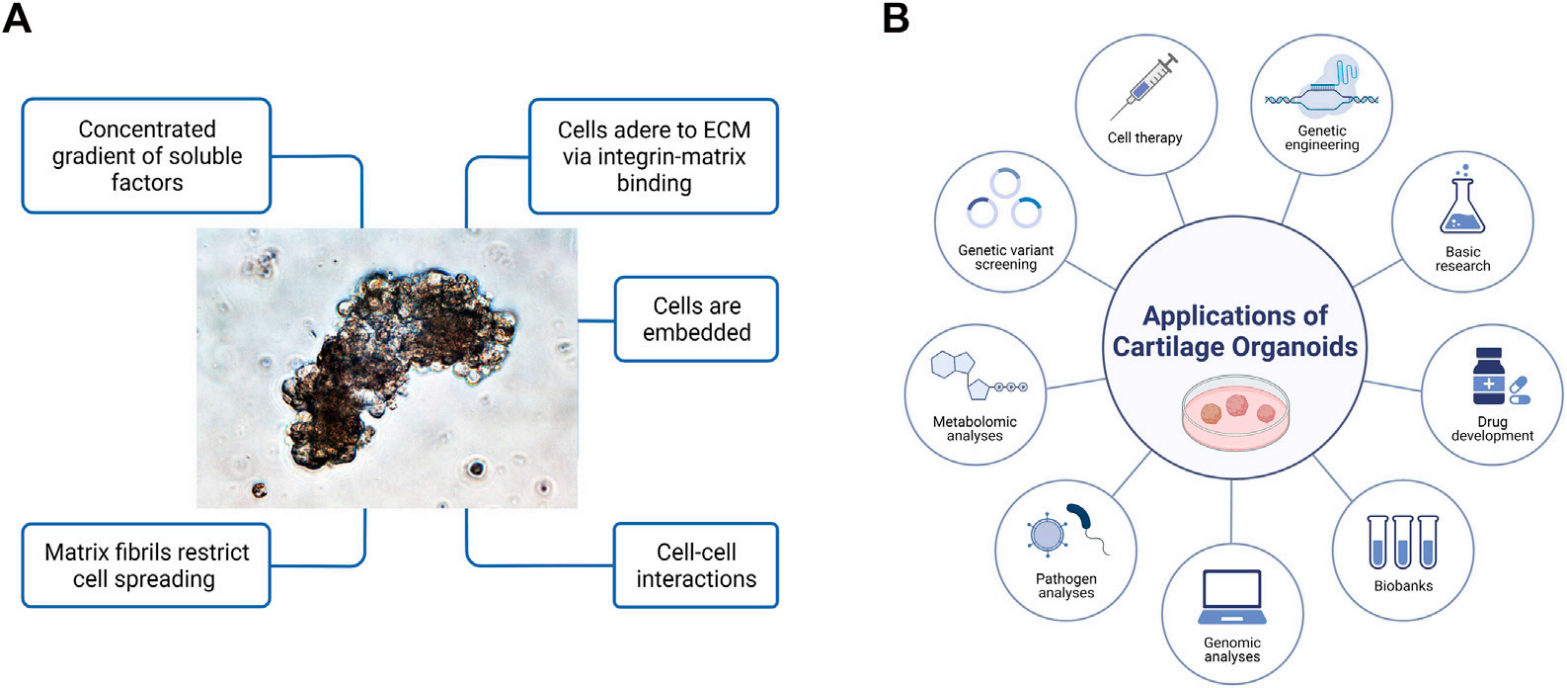
**(A)** General features of three-dimensional cartilage organoids ‘in a dish’. Cells embedded within gels concentrated with various gradients or soluble growth factors are able to adhere to extracellular matrix (ECM), spread and grow with cell-cell interactions in 3-dimensional space. **(B)** Diverse applications of cartilage organoids for preclinical research and clinical transformation of personalized medicine. Cartilage organoid-based implications mainly include cell therapy through multiple functional cell clusters, drug development, genetic engineering, biobanking, genomic analysis, pathogen analysis, metabolomic analysis, and basic preclinical research.

**FIGURE 4:**
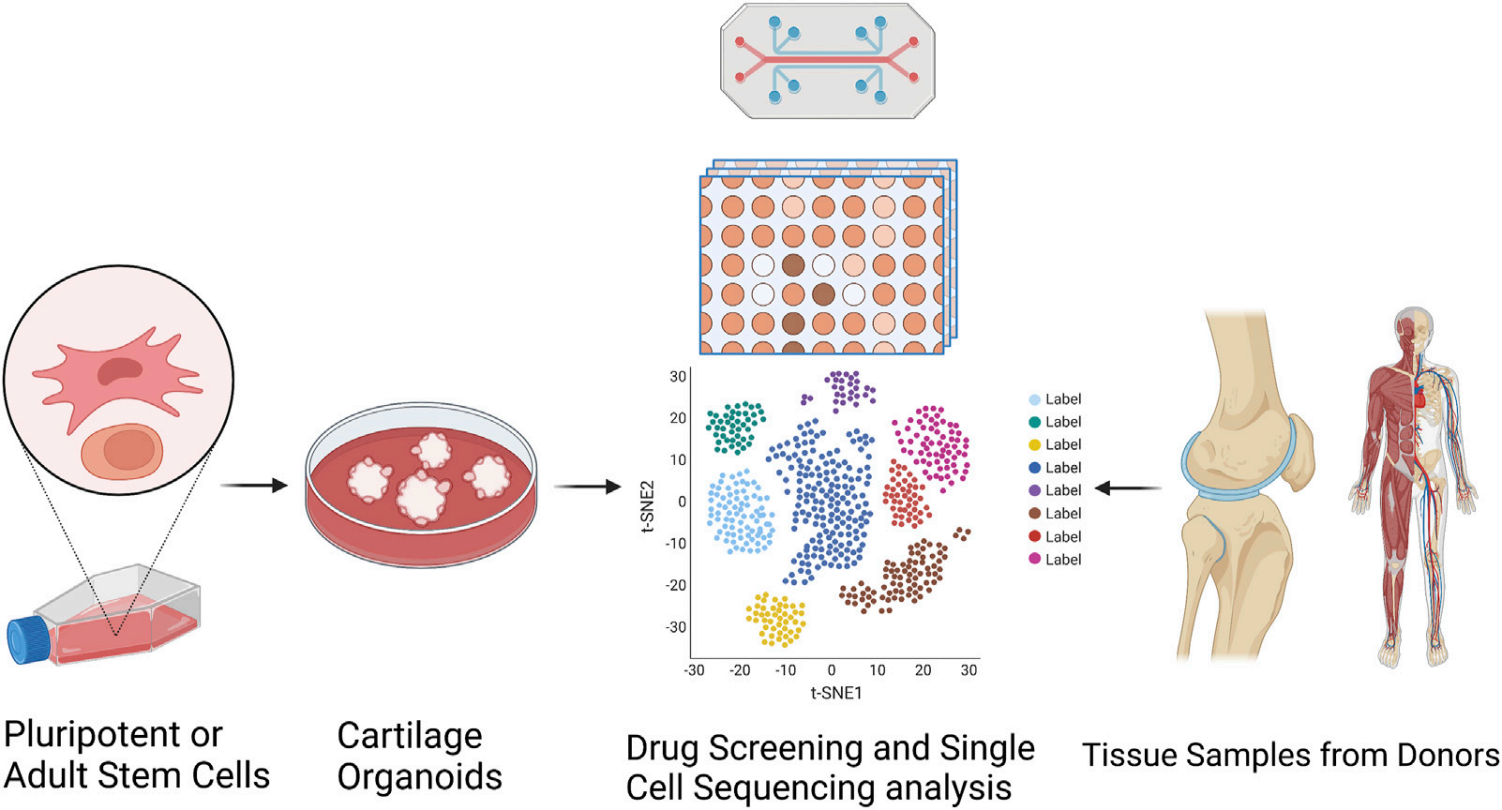
Establishment of cartilage organoid cell atlas through RNA sequencing-based drug discovery and single cell multi-omics analysis. Targeted organoid sequencing through a high-throughput, high-content drug discovery platform targeting RNA-seq to monitor the expression of large gene signatures for the detailed evaluation of cellular phenotypes in cartilage organoids generated from pluripotent or adult stem cells.

**FIGURE 5:**
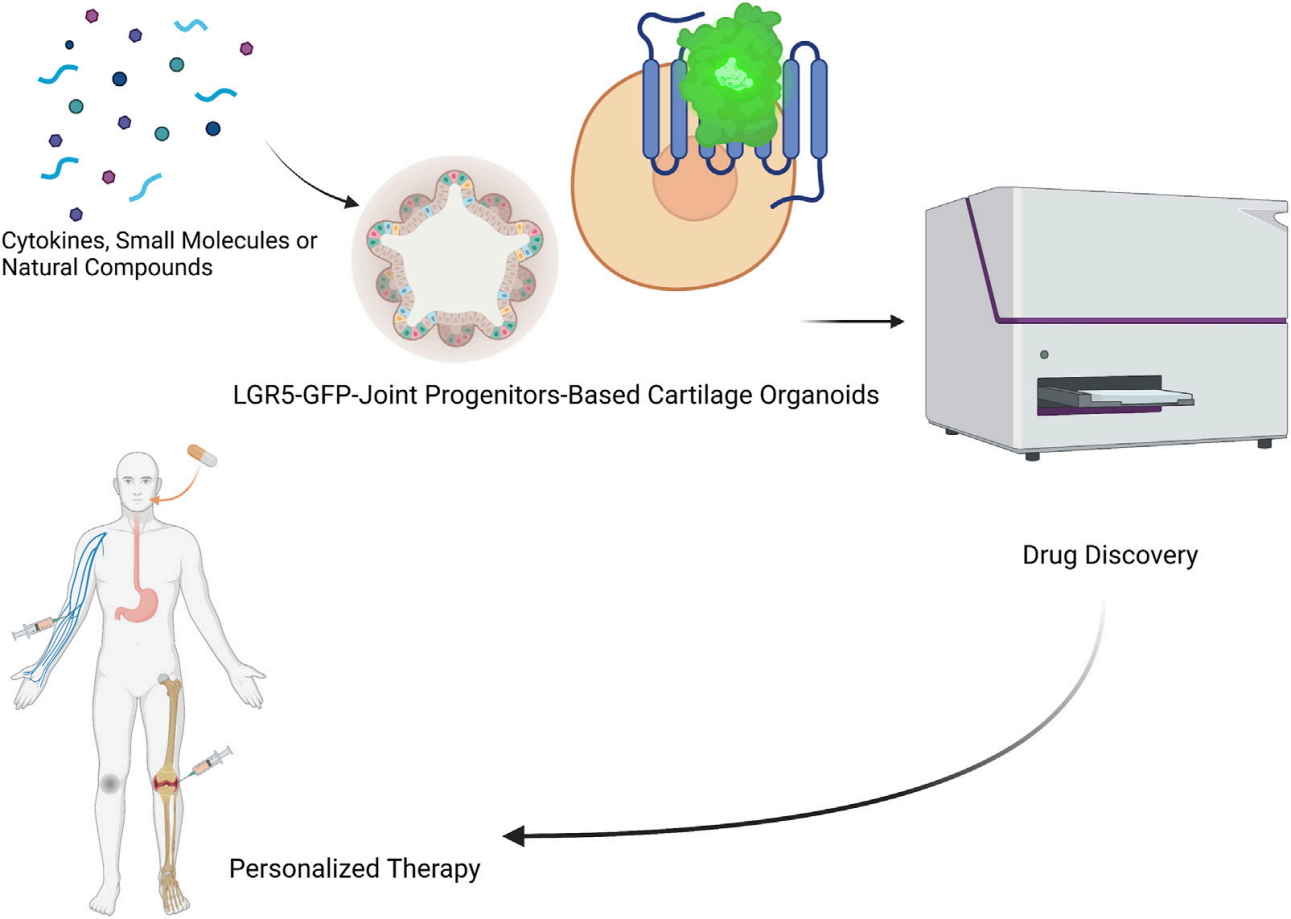
LGR5-joint progenitors-based cartilage organoids for realization of novel drug discovery (identification of novel cytokines, small molecules, and natural compounds), and personalized regenerative therapy of cartilage repair.

Organoids have been firmly established as a robust platform to investigate organ development, normal and pathological processes, and drug screening in both basic preclinical science and translational research, to overcome the limitations associated with animal models (Singh et al., 2021). Optimization of superior cell source, and *ex vivo* culture conditions for phenotypic control of cartilage organoids after transplantation deserve further exploitation. Integrated with advanced technologies (such as 3D bioprinting, bio-assembly, and organ-on-chip-based models, and comprehensive in-depth organoid single-cell genomic atlas mapping through high-spatial-resolution multi-omics sequencing), cartilage organoid models may provide novel molecular, spatial, and temporal insights of embryonic joint development, and (patho)-physiology of cartilage-associated diseases for boosting the development of personalized regenerative therapy for treating cartilage-associated diseases (Liu et al., 2020; Singh et al., 2021). Cartilage organoids-based research on basic preclinical study and clinical transformation of personalized regenerative therapy will put forward a new era of regeneration medicine (Figure 3). Cartilage organoids provide an ideal platform for mechanistic biology at scale for establishment of cartilage organoid cell atlas through high-throughput drug screening or tissue-on-a-chip systems with molecular and phenotypic readout, and single cell multi-omics analysis (Figure 4; Figure 5). Collaborations among bioengineers, pharmacologists, clinicians, and developmental biologists, integrated with cutting-edge technologies and multi-disciplinary platforms, may accelerate the pace of discovery and precision of future clinical translation based on preclinical models of cartilage organoids (Li and Izpisua Belmonte, 2019; Xinaris, 2019; Berishvili et al., 2021; Bhamidipati and Wei, 2022).

## Data Availability

The original contributions presented in the study are included in the article/supplementary files, further inquiries can be directed to the corresponding authors.
